# Video-Assisted Thoracoscopic Decortication of Left Lung Empyema in a Patient With Kartagener's Syndrome

**DOI:** 10.7759/cureus.19281

**Published:** 2021-11-05

**Authors:** Aizaz Khalid, Ali Raza Khan

**Affiliations:** 1 Surgery, Hameed Latif Hospital, Lahore, PAK

**Keywords:** kartagener's syndrome, cystic bronchiectasis, situs inversus with dextrocardia, pleural empyema, video-assisted thoracoscopic surgery (vats), primary ciliary dyskinesia

## Abstract

Kartagener's syndrome is a rare autosomal recessive disorder characterized by the situs inversus, bronchiectasis, and chronic sinusitis. It is found in about half of the individuals with primary ciliary dyskinesia, a disorder of dynein arms in the cilia which renders the mucociliary apparatus inefficient. One of the manifestations of this disorder is the inability to clear secretions from the respiratory pathway leading to recurrent infections and their complications. We present a case of a 16-year-old female with the classical triad of Kartagener's syndrome who developed left-sided empyema thoracis and needed video-assisted thoracoscopic decortication for her condition.

## Introduction

Primary ciliary dyskinesia is a rare condition characterized by defective mucociliary apparatus which predisposes to several systemic manifestations such as bronchiectasis, chronic sinusitis, and subfertility [1]. Approximately half of these patients have Kartagener's syndrome, which is characterized clinically by bronchiectasis, situs inversus, and chronic sinusitis. We have presented a young female with Kartagener's syndrome who developed left lung empyema and underwent video-assisted thoracoscopic decortication.

## Case presentation

A 16-year-old female presented to our institute with worsening left-sided pleuritic chest pain since one week. The pain was associated with increasing productive cough with thick sputum of greenish-yellow color. It was also associated with Grade 3 exertional dyspnea. Her previous medical history included the history of chest infection three years ago, which resolved with administration of oral antibiotics and recurrent nasal congestion with postnasal drip.

The patient did not have any history of prior surgical intervention or hospital admission.

Examination

Her vitals on admission were as follows: blood pressure, 105/65 mmHg; heart rate, 72 beats/min, and respiratory rate, 17 breaths/min. She was afebrile on the presentation.

Physical examination showed a well-oriented young female with pallor. No clubbing was noted. The respiratory exam showed reduced chest expansion and dull note on percussion over left lung fields. Auscultation revealed bilateral rhonchi with reduced air entry on the left side.

Investigations

Blood investigations showed: leukocytosis, 19 x 10^9/L; microcytic hypochromic anemia, hemoglobin 9.9 g/dL, mean corpuscular volume (MCV), 64, with target cells on peripheral smear; and thrombocytosis, platelets 562 x 10^9/L. The rest of the blood work was unremarkable.

**Figure 1:**
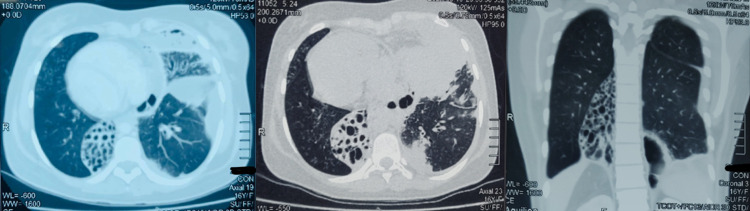
HRCT A (left): Axial view showing right sided cystic bronchiectasis with left sided empyema. B (center): Axial view showing similar finding with empyema extending to anterior mediastinum. C (right): Coronal view showing right sided bronchiectasis with left sided empyema obliterating the costophrenic angle. Note dextrocardia in A and B. HRCT, high resolution computed tomography

A high resolution computed tomography (HRCT) scan was carried out (Figure 1) which showed situs inversus with dextrocardia. It also showed: left lung middle lobe consolidation with empyema; cystic bronchiectasis in posterobasal segment of right lower lobe and bilateral lung infiltrates.

**Figure 2:**
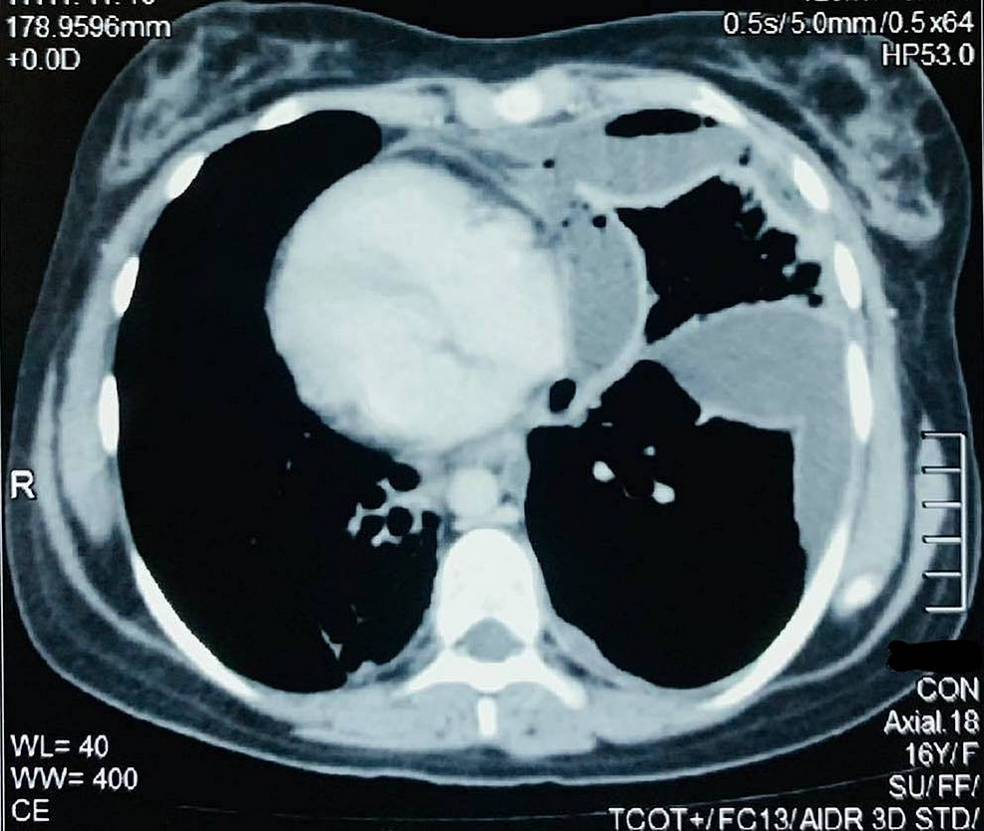
CT chest with IV contrast (axial view) showing dextrocardia and left lung empyema.

This was followed by CT chest with IV contrast (Figure 2) which confirmed the presence of loculated empyema in the left chest.

Management

The patient was diagnosed as a case of Kartagener's syndrome. Initial management was supportive which included bronchodilators, analgesia, chest physiotherapy, and empirical intravenous antibiotics. A thoracic surgery review was sought after a CT scan with IV contrast and it was determined that this patient would benefit from decortication. The patient was then prepared for left-sided video-assisted thoracoscopic decortication.

Bronchoscopy

Preoperative bronchoscopy showed thick secretions in both airways with findings consistent with situs inversus.

Operative findings

**Figure 3:**
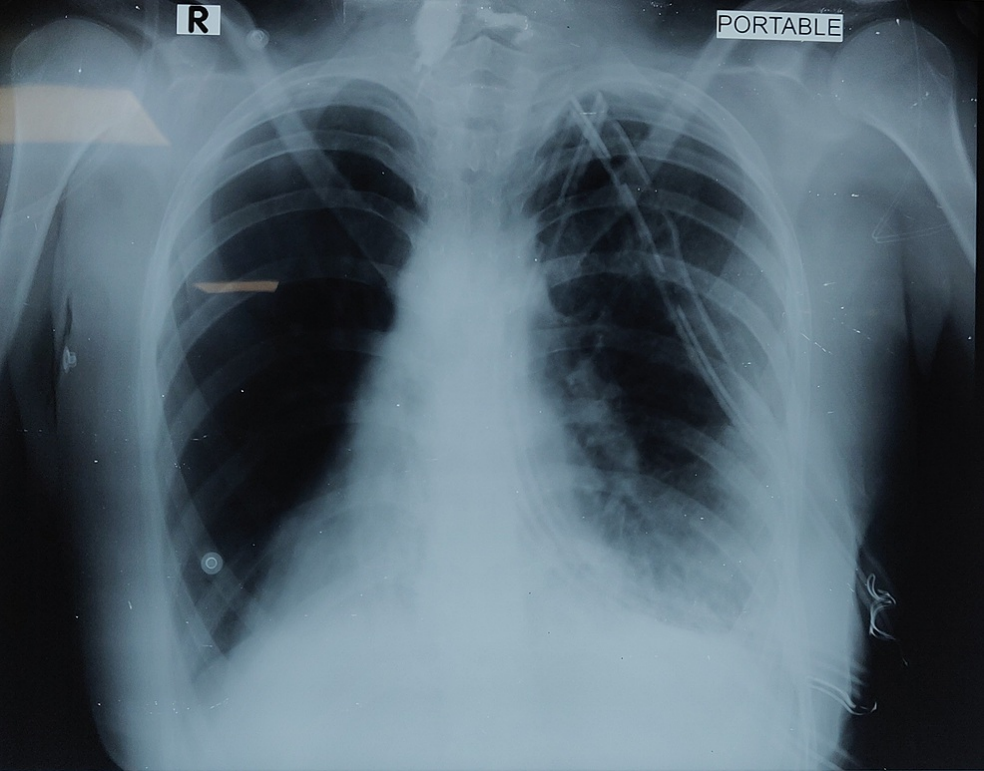
Postoperative chest X-ray.

Left video assisted thoracoscopic surgery (VATS) was carried out by three incisions. Findings consistent with situs inversus were noted. Left lungs showed extensive fibrinous exudate over the middle and lower lobes which was debrided. Pockets of pus were drained after breaking loculations in the anterior mediastinum, oblique fissure, horizontal fissure, and near inferior pulmonary ligament. Satisfactory left lung expansion was noted after decortication. Three 34Fr chest drains were placed and connected to three-chamber chest drainage system on suction. Fluid drained was sent for bacterial, fungal, and tubercular cultures. Postoperative chest X-ray imaging was done (Figure 3).

Postoperative course

The patient was shifted to intensive care unit (ICU) following surgery, where she remained for three days. Her recovery was uneventful with 350 mL drain output on post operative day 1 (POD1), 150 mL on day 2, and 120 mL on day 3. She did not require supplemental oxygen postoperatively. Her pain was managed using epidural infusion of bupivacaine which was discontinued on POD3. Her postoperative care included empirical antibiotics, incentive spirometry, chest physiotherapy, and deep vein thrombosis (DVT) prophylaxis. On the fourth postoperative day her chest tubes were removed and she was discharged after observation.

Follow up

**Figure 4:**
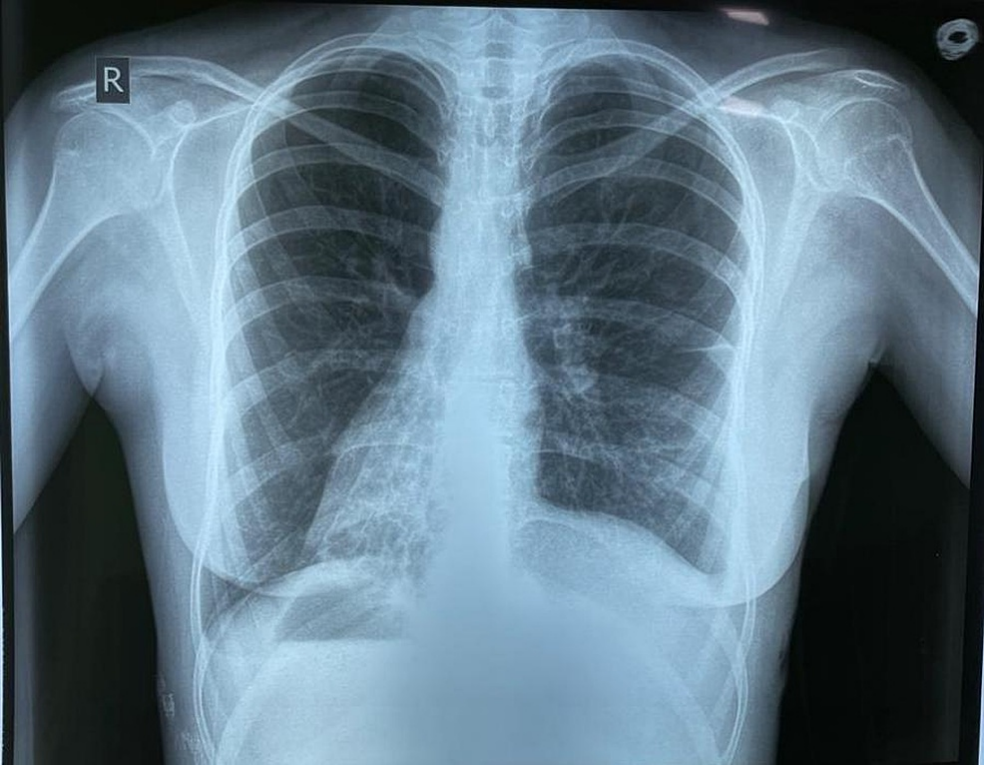
Chest X-ray taken three weeks postoperatively.

The patient was followed up after three weeks in the surgical outdoor. She was vitally stable with no active complaints. She demonstrated complete resolution of her pulmonary symptoms. A follow-up X-ray was also done (Figure 4). The biopsy taken during surgery showed necroinflammatory slough with no growth on fungal/bacterial/TB cultures.

## Discussion

Kartagener's syndrome is a rare autosomal recessive condition characterized by a triad of bronchiectasis, situs inversus, and chronic sinusitis. According to Leigh et al. [1], it is found in about 50% of the subjects with primary ciliary dyskinesia, which is a disorder of ciliary function. The incidence of this primary ciliary dyskinesia (PCD) is estimated to be about one in 30000-60000 with equal prevalence in both genders [2-3].

Cilia in cases with PCD exhibit stiff, uncoordinated, and ineffective movements which is responsible for several clinical manifestations associated with the disease. The pathogenesis of PCD is ultrastructural anomalies in the dynein arms of the cilia which render the ciliary apparatus ineffective [4].

Bronchiectasis is one of the defining features of Kartagener's syndrome and PCD and is associated with a variable decline in respiratory function [5]. The middle and lower lobes are predominantly involved in this disease [6]. Early diagnosis and treatment of this condition is important to prevent and arrest irreversible damage and treatment modalities include chest physiotherapy, bronchodilators, mucolytics, and antibiotics in case of acute exacerbations.

Surgical management of lung disease is generally not recommended due to the generalized nature of this disease. However, lobectomies have shown to improve symptoms in selective patients of PCD with bronchiectasis, including daily cough, hemoptysis, and respiratory infection [7]. Another surgical modality used for patients with end stage lung disease is lung transplantation, which has been successfully carried out in selective cases [8]. However, the long-term benefits of these surgical interventions are largely unknown.

Other manifestations associated with Kartagener's syndrome include chronic sinusitis with chronic otitis media, situs inversus, infertility in males, subfertility in females, clubbing, and telecanthus.

Our patient demonstrated the classical triad associated with Kartagener's syndrome but was unaware of her diagnosis at the time of admission. Delay in the diagnosis of patients with this condition can be a predisposing factor to developing complications such as empyema. Furthermore, video-assisted decortication was an effective treatment for the resolution of symptoms in our patient.

## Conclusions

Kartagener’s syndrome predisposes individuals to the development of pulmonary complications such as bronchiectasis and empyema thoracic. Video-assisted thoracoscopic decortication is an effective approach for the management of empyema thoracis in patients with Kartagener's syndrome. Further studies are required on this subject to improve case-specific management guidelines for treatment as well as prevention of complications in patients with primary ciliary dyskinesia and Kartagener's syndrome.
